# Distribution of a novel *CYP2C* haplotype in Native American populations

**DOI:** 10.3389/fgene.2023.1114742

**Published:** 2023-03-21

**Authors:** Vanessa Câmara Fernandes, Marco Antônio M. Pretti, Luiza Tamie Tsuneto, Maria Luiza Petzl-Erler, Guilherme Suarez-Kurtz

**Affiliations:** ^1^ Coordenação de Pesquisa, Instituto Nacional de Câncer, Rio de Janeiro, Brazil; ^2^ Laboratório de Bioinformática e Biologia Computacional, Instituto Nacional de Câncer, Rio de Janeiro, Brazil; ^3^ Departamento de Análises Clínicas, Universidade Estadual de Maringá, Maringá, Brazil; ^4^ Programa de Pós-Graduação em Genética, Departamento de Genética, Universidade Federal do Paraná, Curitiba, Brazil

**Keywords:** amerindians, CYP2C cluster, CYP2C19 metabolic phenotypes, native American populations, pharmacogenetics

## Abstract

The *CYP2C19* gene, located in the *CYP2C* cluster, encodes the major drug metabolism enzyme CYP2C19. This gene is highly polymorphic and no-function (CYP2C19*2 and CYP2C19*3), reduced function (CYP2C19*9) and increased function (CYP2C19*17) star alleles (haplotypes) are commonly used to predict CYP2C19 metabolic phenotypes. CYP2C19*17 and the genotype-predicted rapid (RM) and ultrarapid (UM) CYP2C19 metabolic phenotypes are absent or rare in several Native American populations. However, discordance between genotype-predicted and pharmacokinetically determined CYP2C19 phenotypes in Native American cohorts have been reported. Recently, a haplotype defined by rs2860840T and rs11188059G alleles in the *CYP2C* cluster has been shown to encode increased rate of metabolism of the CYP2C19 substrate escitalopram, to a similar extent as CYP2C19*17. We investigated the distribution of the *CYP2C:TG* haplotype and explored its potential impact on CYP2C19 metabolic activity in Native American populations. The study cohorts included individuals from the One Thousand Genomes Project AMR superpopulation (1 KG_AMR), the Human Genome Diversity Project (HGDP), and from indigenous populations living in Brazil (Kaingang and Guarani). The frequency range of the *CYP2C:TG* haplotype in the study cohorts, 0.469 to 0.598, is considerably higher than in all 1 KG superpopulations (range: 0.014—to 0.340). We suggest that the high frequency of the *CYP2C:TG* haplotype might contribute to the reported discordance between *CYP2C19-*predicted and pharmacokinetically verified CYP2C19 metabolic phenotypes in Native American cohorts. However, functional studies involving genotypic correlations with pharmacokinetic parameters are warranted to ascertain the importance of the *CYP2C:TG* haplotype.

## Introduction

The human CYP2C subfamily comprises four members, namely, CYP2C18, CYP2C19, CYP2C9 and CYP2C8, with encoding genes located in tandem in chromosome 10q23-24. CYP2C19 provides the major pathway for biotransformation of a variety of drugs from different therapeutic classes, including antidepressants, both tricyclic (e.g., imipramine) and selective serotonin reuptake inhibitors (escitalopram), antifungal (voriconazole), antimalarial (proguanil) and antiplatelet (clopidogrel) drugs, and proton pump inhibitors (omeprazole). CYP2C19-mediated metabolism may lead to drug inactivation (e.g., omeprazole and voriconazole) as well as to active metabolites, which account for the clinical effects of pro-drugs such as proguanil and clopidogrel (Botton et al., 2021). The clinical relevance of the CYP2C19 pathway is reflected in the CPIC (Clinical Pharmacogenetics Implementation Consortium) guidelines: five of the 26 guidelines currently available have dosing recommendations based on CYP2C19 metabolic phenotypes, predicted from *CYP2C19* genotypes (Hicks et al., 2015, 2017; Moriyama et al., 2017; Lima et al., 2021; Lee et al., 2022).

The *CYP2C19* gene is highly polymorphic, with 39 star alleles (haplotypes) currently defined in the Pharmacogene Variation Consortium (https://www.pharmvar.org/gene/CYP2C19). Five star alleles, namely, CYP2C19*2 and *3 (no-function alleles), CYP2C19*9 (reduced function) CYP2C19*17 (increased function) plus the default wildtype allele (CYP2C19*1) are used in the CPIC guidelines to predict CYP2C19 metabolic phenotypes. However, a novel *CYP2C* haplotype, comprising the rs2860840T (*CYP2C18*, 3’ UTR) and rs11188059G (*CYP2C18*, intron 5) alleles in the *CYP2C* cluster has been recently shown to encode increased rate of metabolism of the CYP2C19 substrate escitalopram to at least a similar extent as CYP2C19*17 (Bråten et al., 2021).

The present study investigates the distribution of the *CYP2C:TG* haplotype and its potential impact on prediction of CYP2C19 metabolic phenotypes in Native American populations. Previous studies have shown that the CYP2C9*17 and, consequently, the genotype-assigned rapid (RM) and ultrarapid (UM) CYP2C19 metabolic phenotypes, are absent or rare in Native Americans (Vargens et al., 2012; Bonifaz-Peña et al., 2014; de Andrés et al., 2021, 2017; Naranjo et al., 2018; Rodrigues Soares et al., 2020; de Andrés et al., 2021). However, discordance between genotype-predicted and pharmacokinetically determined CYP2C19 phenotypes in Native American cohorts have been reported, such that individuals genotyped as CYP2C19*1/*1 and assigned the normal metabolic (NM) phenotype showed greater CYP2C19 activity than UMs (de Andrés et al., 2017).

## Methods

### Study populations

Four cohorts were investigated, namely, 1 KG_NAT - a sub-cohort of the One Thousand Genomes Project Admixed American superpopulation (denoted 1 KG_AMR; Auton et al., 2015) -, HGDP Native Americans (Cavalli-Sforza, 2005), and Kaingang and Guarani living in Brazil (Petzl-Erler et al., 1993; Tsuneto et al., 2003). The 1 KG AMR comprises individuals from the South American countries Colombia (denoted CLM) and Peru (PEL), from Puerto Rico (PUR) as well as people of Mexican Ancestry (MXL) living in Los Angeles, United States of America. The 1 KG_NAT comprised the 68 1 KG_AMR individuals (58 PEL and 10 MXL) with the highest proportions of Native ancestry: median 94.5%, IQR 85.7%–100% (Suarez-Kurtz et al., 2020). The HGDP cohort (n = 61) is formed by samples of Native American groups, from Brazil (Surui and Karitiana), Mexico (Maya and Pima) and Colombia. Kaingang (KRC) and Guarani (GRC and GKW) are represented by adults enrolled in a population genetics study of Brazilian Amerindians, approved by the Brazilian National Ethics Committee (CONEP123/98). Kaingang and Guarani, the two major Amerindian tribes of southern Brazil, are culturally quite distinct from each other, the Guarani belonging to the Tupi linguistic group, while Kaingang are Gê-speaking. The KRC and GRC live in different villages within the Rio das Cobras reservation (25º18′S, 52º32′W), whereas GKW are from the Amambai and Limão Verde reservations (23º06′S, 55º12′W and 23º12′S, 55º06′W, respectively).

### *CYP2C19* and *CYP2C* alleles and haplotypes

We analyzed six single nucleotide polymorphisms (SNPs) in the *CYP2C* cluster, namely, rs2860840C>T (GRCh38.13 chr 10:94735475) and rs11188059G>A (GRCh38.13 chr 10:94709142) in *CYP2C18*, and rs4244285G>A (GRCh38.13 chr 10:94781859, CYP2C19*2), rs4986893G>A (GRCh38.13 chr 10:94780653, CYP2C19*3), rs17884712G>A (GRCh38.13 chr 10:94775489, CYP2C19*9), rs12248560C>T (GRCh38.13 chr 10:94761900, CYP2C19*17) in the *CYP2C19* gene*.* Genotype data from 1 KG_AMR were retrieved at https://www.ensembl.org/index.html whereas aligned sequences for the 61 individuals from the Human Genome Diversity Project (HGDP) were retrieved at http://ftp.1000genomes.ebi.ac.uk/vol1/ftp/data_collections/HGDP/. The Kaingang and Guarani samples were genotyped using a 7500 Real-Time System and TaqMan allele discrimination probes for rs2860840 (C_11201742_10), rs11188059 (C_31983321_10), rs4244285 (C_25986767_70) and rs12248560 (C_469,857_10), according to the manufacturer’s instructions. The CYP2C19*1 (wild-type allele) was assigned by default, i.e., absence of variant alleles at the *CYP2C19* loci genotyped.

Individual haplotypes and diplotypes were inferred using the HaploStats software implemented on the R platform. This software attributes a posterior probability value for the diplotype configuration for each individual on the basis of estimated haplotype frequencies. The minimal posterior probability value for inclusion of an individual in these analyses was set at 0.95. We adopted the labelling used by Bråten *et al.* (2021) to denote *CYP2C* haplotypes and diplotypes comprising the *CYP2C18* rs2860840 and rs11188059 SNPs.

### Assignment of CYP2C19 metabolic phenotypes

CYP2C19 metabolic phenotypes were assigned according to either the *CYP2C19* guidelines (Hicks et al., 2015, 2017; Moriyama et al., 2017; Lima et al., 2021; Lee et al., 2022), or as proposed by Bråten *et al.* (2021).

### Statistical analyses

Deviation of genotype distribution from Hardy-Weinberg equilibrium was assessed by the goodness-of-fit χ^2^ test. Chi square tests were applied to compare the distribution of *CYP2C* haplotypes and predicted CYP2C19 across cohorts. Significance level was set at *p* < 0.05.

## Results and discussion

There were no significant deviations from Hardy-Weinberg equilibrium at the *CYP2C18* and *CYP2C19* loci interrogated. The two *CYP2C18* SNPs were common in all study cohorts (Table 1): the minor allele frequency (MAF) of rs11188059G>A ranged from 0.107 (HGDP) to 0.481 (Kaingang), whereas the frequency of the variant rs2860840 T allele reached 0.963 in Kaingang, and ranged between 0.705–0.757 in the other cohorts. These results are consistent with data for North and South American Native populations in the Allele Frequency Database (https://alfred.med.yale.edu/).

**TABLE 1:** Distribution of *CYP2C18* alleles and *CYP2C* diplotypes in Native Americans.

Cohorts (n individuals)	1 KG_NAT (68)	HGDP (61)	Kaingang (54)	Guarani (33)
***CYP2C18* SNPs**	Variant allele frequency			
rs2860840 C>T	0.757	0.705	0.963	0.727
rs11188059 G>A	0.265	0.107	0.481	0.242
***CYP2C* diplotypes** ^#^	Diplotype frequency			
TG	0.493	0.598	0.490	0.469
TA	0.265	0.107	0.471	0.250
CG	0.243	0.295	0.038	0.281
CA	0	0	0	0

^#^ comprising rs2860840 C>T and rs11188059 G>A, and denoted as in Bråten et al. (2021)

Three haplotypes comprising rs2860840C>T and rs11188059G>A (*CYP2C* haplotypes) were identified in the study cohorts (Table 1): *CYP2C:TG* was the most common, with frequencies ranging from 0.469 to 0.598, while *CYP2C:CG* and *CYP2C:TA* frequencies ranged between 0.038–0.295 and 0.107–0.471, respectively. The frequencies of these *CYP2C* haplotypes in the study cohorts differed markedly from the African (1 KG_AFR), European (1 KG_EUR), East Asian (1 KG_EAS) and South-Asian (1 KG_SAS) 1 KG superpopulations (Supplementary Table S1): *CYP2C:TG* and, to a lesser extent, *CYP2C:TA* were more common in the study cohorts than in the 1 KG superpopulations, whereas the opposite was observed for *CYP2C:CG*. The *CYP2C:CA* haplotype, absent or extremely rare in the 1 KG superpopulations was absent in the Native American cohorts of our study. Pairwise comparisons of the frequency of *CYP2C:TG, TA* and *CG* haplotypes in any study cohort *versus* any *1 KG* superpopulation disclosed highly significant differences (chi square *p* < 0.0001).

The high frequency of *CYP2C:TG* in the study cohorts was of special interest to us in reference to the reported discordance observed in Native Americans, between CYP2C19 metabolic phenotypes predicted from *CYP2C19* diplotypes *versus* phenotypes determined by pharmacokinetic measurements (de Andrés et al., 2017; Naranjo et al., 2018). For example, de Andrés *et al.* (2017) observed that several Mexican Amerindians genotyped as CYP2C19*1/*1 and assigned the normal metabolizer (NM) phenotype had higher CYP2C19 activity than genotype-predicted ultrarapid metabolizers (UMs), i.e., carriers of the CYP2C19*17/*17 diplotype. We hypothesized that such discrepancy might result from linkage of CYP2C19*1 with the *CYP2C:TG* haplotype, as reported in the pivotal study of Bråten *et al.* (2021).

To explore this hypothesis, we genotyped the *CYP2C19* star alleles *2, *3, *4 and *17 which are used in the CPIC guidelines to predict CYP2C19 metabolic phenotypes. As shown in Table 2, CYP2C19*2 was absent in Kaingang and its MAF ranged between 0.057 and 0.109 in the other three cohorts. CYP2C19*3 and CYP2C19*9 were not detected in 1 KG_NAT and HGDP Native Americans and could not be interrogated in Kaingang and Guarani samples due to the limited amount of DNA available. CYP2C19*17 was absent in HGDP and Kaingang, and had MAF of 0.022 in 1 KG_NAT and Guarani. These data are consistent with previous studies in other Native American groups (Vargens et al., 2012; Bonifaz-Peña et al., 2014; de Andrés et al., 2021, 2017; Naranjo et al., 2018; Rodrigues Soares et al., 2020). For example, Rodrigues-Soares et al. (2020) showed that CYP2C19*2 is absent in Guaymi from Costa Rica and Tzeltal from Mexico, CYP2C19*17 is absent in various Mayan and Uto-Aztecan groups from Mexico, as well as from Arawak and Quechuamara from Peru, while CYP2C19*3 is not detected in the vast majority of Native American populations, including a Guarani cohort previously studied by our group (Vargens et al., 2012).

**TABLE 2:** Distribution of *CYP2C19* alleles, diplotypes and assigned phenotypes.

Cohorts (n individuals)		1 KG_NAT (68)	HGDP (61)	Kaingang (54)	Guarani (33)
**SNPs (star alleles)**		Allele frequency			
rs4244285 G>A (CYP2C19*2)		0.066	0.057	0	0.109
rs4986893 G>A (CYP2C19*3)		0	0	ng	ng
rs28399504 A>G (CYP2C19*9)		0	0	ng	ng
rs12248560 C>T (CYP2C19*17)		0.022	0	0	0.018
**Diplotypes**	Phenotypes^#^	Diplotype frquency			
*1/*1	NM	0.838	0.885	1.0	0.758
*1/*2	IM	0.118	0.115	0	0.212
*2/17	IM	0.015	0	0	0
*1/*17	RM	0.029	0	0	0.030

^#^ assigned according to the CPIC guidelines [2–6]. NM, normal metabolizer; IM, intermediate metabolizer; RM, rapid metabolizer.

We then examined the distribution of *CYP2C19* diplotypes and CYP2C19 phenotypes assigned according to the CPIC guidelines (Table 2): All Kaingang individuals were homozygous for the default CYP2C19*1 allele and therefore assigned the NM phenotype. In the 1 KG_NAT, HGDP and Guarani cohorts, wildtype homozygosis (CYP2C19*1/*1; NM phenotype) ranged from 0.758 to 0.885, while other diplotypes and assigned phenotypes were distributed as follows: CYP2C19*1/*2 (IM phenotype) ranged from 0.118, in 1 KG_NAT and HGDP, to 0.212, in Guarani; CYP2C19*2/*17 (IM) was detected in only one 1 KG_NAT individual (0.015); CYP2C19*1/*17 (RM phenotype) was absent in HGDP and rare (0.03) in 1 KG_NAT and Guarani; homozygous CYP2C19*2/*2 (PM) and CYP2C19*17/*17 (UM phenotype*)* were not detected.

**FIGURE 1:**
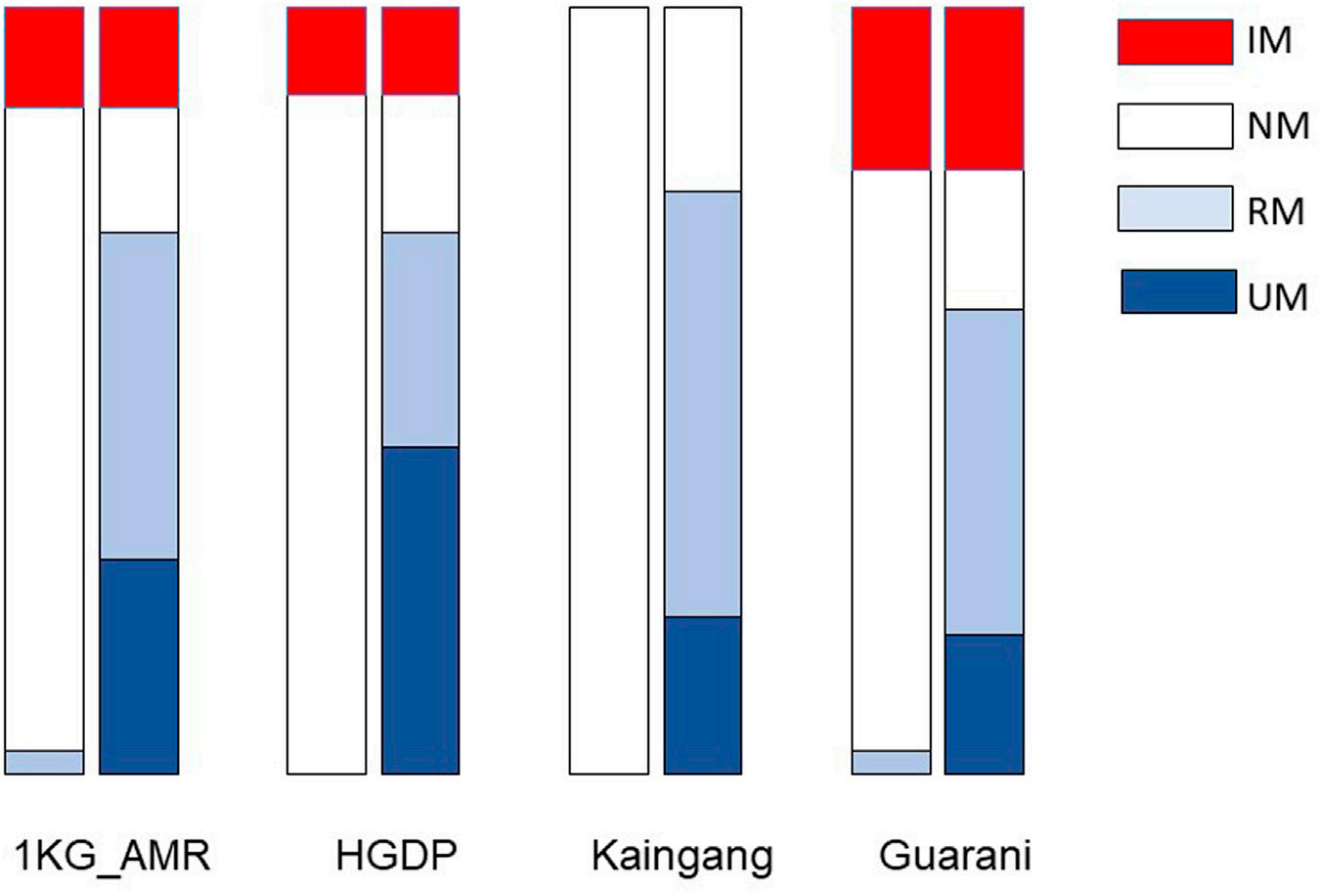
Distribution of predicted CYP2C19 metabolic phenotypes in the study cohorts. In each pair of columns, the left column shows phenotype prediction based on *CYP2C19* diplotypes (Table 2) and the right column shows phenotype prediction based on *CYP2C19-CYP2C* diplotypes (Table 3). The two assignment procedures resulted in highly significant differences in phenotype distribution in all cohorts (chi-square *p* < 0.0001). NM, normal metabolizer; RM, rapid metabolizer; UM, ultrarapid metabolizer; IM intermediate metabolizer.

Next, we applied the Haplo-Stats software to infer the individual haplotypes and diplotypes formed by the *CYP2C19* star alleles and the *CYP2C* diplotypes (Table 3). Five haplotypes were identified, of which three had the wild-type CYP2C19*1 linked to one of *CYP2C:CG* (denoted haplotype *1CG)*, CYP2C:TA* (*1 TA) or CYP2C:TG (*1 TG); the two other haplotypes were formed by *CYP2C:CG* linked to either CYP2C19*2 (*2CG) or CYP2C19*17 (*17CG). The *1 TG haplotype was the most frequent in all cohorts (range 0.470–0.598). While the *1CG and *1 TA haplotypes ranged in frequency between 0.037–0.238 and 0.107–0.481, respectively of notice, the *CYP2C:TG* haplotype was never linked to either rs4244285 A (CYP2C19*2) or rs12248560 T (CYP2C19*17) variant alleles, in concordance with the observation of Bråten *et al.* (2021), that *CYP2C:TG* is in “complete linkage disequilibrium with the c.991A>G (I331V; CYP2C19*1.002) variant, like the majority of CYP2C19*1-alleles”. Also, following these authors’ approach, the *CYP2C:CG* and *CYP2C:TA* haplotypes were merged for statistical analyses of the distribution of *CYP2C19:CYP2C* diplotypes and assigned metabolic phenotypes (Table 3). Eight diplotypes were observed, leading to assignment of NM, IM, RM and UM phenotypes. For visual comparison, plots of the distribution of CYP2C19 metabolic phenotypes predicted according to the *CYP2C19* (Table 2) or the *CYP2C19-CYP2C* diplotypes (Table 3) are shown in Figure 1. The two assignment procedures resulted in highly significant differences in phenotype distribution in all cohorts (*p* < 0.0001). The overall picture is the unveiling of UMs and large increases in frequency of RMs at the expense of NM, with no impact on IM frequency or on the absence of PMs when the *CYP2C19-CYP2C* diplotypes are used for phenotype assignment. This was observed in all cohorts, and most flagrant in Kaingang: the absence of the CYP2C19*2 or CYP2C19*17 alleles leads to assignment of the NM phenotype to all Kaingang individuals, according to *CYP2C19* diplotypes. By contrast, when phenotype assignment is based on *CYP2C19-CYP2C* diplotypes, NMs represent only 24% of the cohort, while RMs and UMs account for 56% and 20%, respectively.

**TABLE 3:** Distribution of *CYP2C19-CYP2C* diplotypes and assigned CYP2C19 phenotypes.

*CYP2C19-CYP2C*		1 KG_NAT (68) ^#^	HGDP (61)	Kaingang (54)	Guarani (33)
**Haplotype**		Haplotype frequency			
*1CG		0.154	0.238	0.037	0.081
*1 TA		0.265	0.107	0.481	0.167
*1 TG		0.493	0.598	0.481	0.470
*2CG		0.066	0.057	0	0.106
*17CG		0.022	0	0	0.015
**Diplotype**	**Assigned phenotype** ^##^	Diplotype frequency			
*1CG or TA/*1CG or TA	NM	0.162	0.180	0.241	0.182
*1CG or TA/*1TG	RM	0.426	0.279	0.556	0.394
*1TG/*1TG	UM	0.250	0.426	0.204	0.182
*1CG or TA/*2CG	IM	0.088	0.049	0	0.030
*1TG/*2CG	IM	0.029	0.066	0	0.182
*1CG or TA/*17CG	RM	0	0	0	0.030
*1TG/*17CG	UM	0.029	0	0	0
*2CG/*17CG	IM	0.015	0	0	0

^#^ number of individuals in brackets.

^##^ diplotypes were denoted and phenotypes were assigned as proposed by Bråten et al. (2021).

NM, normal metabolizer; RM, rapid metabolizer, UM, ultrarapid metabolizer; IM, intermediate metabolizer; PM, poor metabolizer.

The fact that all study individuals with *CYP2C19:CYP2C*-predicted RM or UM phenotypes carry the CYP2C19*1/*1 diplotype might possibly offer an explanation for the reported discordance, alluded above, between *CYP2C19*-predicted and pharmacokinetically-verified phenotypes in Native Americans (de Andrés et al., 2017; Naranjo et al., 2018). However, we are fully aware that functional studies involving genotypic correlations with pharmacokinetic parameters are warranted to validate this suggestion. Modulation of CYP2C19 activity by the *CYP2C:TG* genotype was first observed in relation to escitalopram disposition in a cohort of predominantly “White origin” (Bråten et al., 2021), and was subsequently associated with failure of omeprazole treatment of New Zealand European GERD (gastroesophageal reflux disease) patients (Kee et al., 2022). There is no comparative data in Native American populations. The mechanism whereby the *CYP2C:TG* haplotype may increase CYP2C19-dependent metabolism needs to be addressed further. Bråten *et al.* (2021) suggested tentatively that the rs2860840 T allele “has a functional role as increasing the enhancer function and *CYP2C19* expression” whereas the rs11188059 A variant abolishes this effect, such that the *CYP2C:TG*-haplotype, but not the *CYP2C:TA* haplotype associates with increased CYP2C19 activity. There is prior evidence for long-range haplotypes across the *CYP2C* cluster, that may form functional units, notably one defined by rs12777823, an intergenic polymorphism reported to be strongly associated with requirement of reduced warfarin doses among African Americans and black Africans (Perera et al., 2013; Ndadza et al., 2019).

We acknowledge the low number of individuals of distinct groups in the HGDP and Guarani cohorts as a limitation of our study. Practical and ethical difficulties are commonly encountered in recruiting participants from Native American populations, such that in a recent overview of the distribution of *CYP2C19* variants and predicted phenotypes among Native American groups, 9 out of the 19 studied cohorts had less than 50 individuals (Rodrigues-Soares et al., 2020). In addition, we caution that the present data should not be interpreted as representative of all extant Amerindian populations, in view of their high level of (pharmaco)genetic diversity (Gaspar et al., 2002; Suarez-Kurtz et al., 2019; Fernandes et al., 2022).

## Data Availability

The original contributions presented in the study are included in the article/Supplementary Material, further inquiries can be directed to the corresponding author.
