# Supplementary material for: Distribution of a novel CYP2C haplotype in Native American populations
Source: Front Genet. 2023 Mar 21;14:1114742. doi: 10.3389/fgene.2023.1114742 (PMC10071019; doi:10.3389/fgene.2023.1114742)
Supplement: Supplementary file 1 [file Table1.DOCX]

| **Supplementary Table 1. Distribution of *CYP2C18* diplotypes in 1KG superpopulations** | | | | | | |
| --- | --- | --- | --- | --- | --- | --- |
| *CYP2C* | 1000 Genomes Project superpopulations | | | | | |
| diplotypes^+^ | AFR (1320)^++^ | AMR (694) | EAS (1008) | EUR (1006) | SAS (978) | |
| CG | 0.979 | 0.486 | 0.831 | 0.664 | 0.766 | |
| TG | 0.014 | 0.340 | 0.166 | 0.202 | 0.144 | |
| TA | 0.007 | 0.174 | 0.002 | 0.128 | 0.089 | |
| CA | 0 | 0 | 0.001 | 0 | 0.001 | |
| Data presented as proportion of the *CYP2C* diplotypes in each 1KG superpopulation: AFR, Africans; AMR, Admixed Americans; EAS, East Asians; EUR, Europeans; SAS, South Asians.  ^+^  diplotypes of rs2860840C>T and rs11188059G>A provided by <https://ldlink.nci.nih.gov/>  ^++^ number of chromosomes in brackets | | | | | |  |
